# *N*-(4-Chloro­benzo­yl)-*N*′-(3-fluoro­phen­yl)thio­urea

**DOI:** 10.1107/S1600536810030965

**Published:** 2010-08-11

**Authors:** Nur Eliyanti A. Othman, M. Ibrahim M. Tahir, Bohari M. Yamin

**Affiliations:** a School of Chemical Sciences and Food Technology, Universiti Kebangsaan Malaysia, UKM 43500 Bangi Selangor, Malaysia; b Department of Chemistry, Faculty of Science, Universiti Putra Malaysia, 43400 UPM Serdang, Selangor, Malaysia

## Abstract

In the title compound, C_14_H_10_ClFN_2_OS, the mol­ecule adopts a *trans*–*cis* geometry of the thio­urea unit. The dihedral angles between the benzene rings is 34.47 (7)°. The crystal packing features inter­molecular N—H⋯S and C—H⋯O hydrogen bonds, forming a chain along the *b* axis. A short C—H⋯S intramolecular contact is also observed.

## Related literature

For the biological and anti corrosion properties of thio­urea derivatives, see: Shen *et al.* (2006 ▶); Sun *et al.*(2006 ▶). For the structures of related 4-chloro­benzoyl thio­urea derivatives, see: Khawar Rauf *et al.* (2009 ▶); Yusof *et al.* (2009 ▶). For bond-length data, see: Allen *et al.* (1987 ▶).
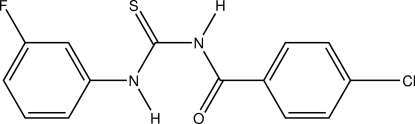


## Experimental

### 

#### Crystal data

C_14_H_10_ClFN_2_OS
*M* *_r_* = 308.75
Monoclinic, 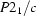

*a* = 8.5778 (1) Å
*b* = 11.7584 (2) Å
*c* = 13.4069 (2) Å
β = 92.448 (2)°
*V* = 1351.00 (3) Å^3^
*Z* = 4
Cu *K*α radiation
μ = 4.03 mm^−1^
*T* = 293 K
0.50 × 0.29 × 0.25 mm

#### Data collection

Oxford Diffraction Xcalibur Eos Gemini diffractometer
Absorption correction: multi-scan (*CrysAlis PRO*; Oxford Diffraction, 2010 ▶) *T*_min_ = 0.238, *T*_max_ = 0.432
33403 measured reflections
2685 independent reflections
2628 reflections with *I* > 2σ(*I*)
*R*_int_ = 0.027

#### Refinement

*R*[*F* ^2^ > 2σ(*F* ^2^)] = 0.032
*wR*(*F* ^2^) = 0.090
*S* = 1.06
2685 reflections
181 parameters
H-atom parameters constrained
Δρ_max_ = 0.31 e Å^−3^
Δρ_min_ = −0.27 e Å^−3^

### 

Data collection: *CrysAlis PRO* (Oxford Diffraction, 2010 ▶); cell refinement: *CrysAlis PRO*; data reduction: *CrysAlis PRO*; program(s) used to solve structure: *SHELXS97* (Sheldrick, 2008 ▶); program(s) used to refine structure: *SHELXL97* (Sheldrick, 2008 ▶); molecular graphics: *SHELXTL* (Sheldrick, 2008 ▶); software used to prepare material for publication: *SHELXTL*, *PARST* (Nardelli, 1995 ▶) and *PLATON* (Spek, 2009 ▶).

## Supplementary Material

Crystal structure: contains datablocks global, I. DOI: 10.1107/S1600536810030965/dn2588sup1.cif

Structure factors: contains datablocks I. DOI: 10.1107/S1600536810030965/dn2588Isup2.hkl

Additional supplementary materials:  crystallographic information; 3D view; checkCIF report

## Figures and Tables

**Table 1:** Hydrogen-bond geometry (Å, °)

*D*—H⋯*A*	*D*—H	H⋯*A*	*D*⋯*A*	*D*—H⋯*A*
C14—H14*A*⋯S1	0.93	2.57	3.1865 (15)	124
N2—H2*A*⋯O1	0.86	1.91	2.6402 (16)	141
N1—H1*A*⋯S1^i^	0.86	2.68	3.4134 (13)	145
C2—H2*B*⋯O1^ii^	0.93	2.48	3.3717 (18)	160

Symmetry codes: (i) 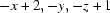; (ii) 
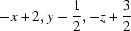
.

**Fig. 1.**
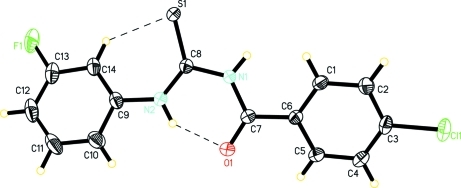
The nolecular structure of (I), with displacement ellipsods drawn at the 50% probability level.

**Fig. 2.**
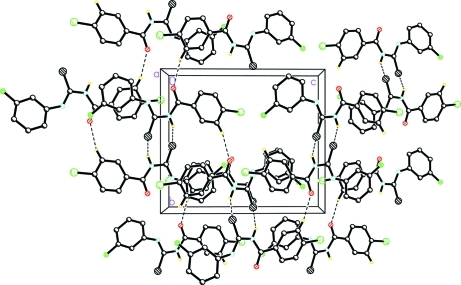
A packing diagram of (I) viewed down the a axis. Hydrogen bonds are shown by dashed lines.

## supplementary crystallographic information

### Comment

The rapid progress in the synthesis of thiourea derivative is driven by their potential as biological active compounds (Sun *et al.*, 2006) and in the material applications such as anti corrosion(Shen *et al.*, 2006). The molecular structural study of the compound is important for structure-activity relationship which is useful for rasional design strategy. The tittle compound (I) is analogus to 1-(4-chlorobenzoyl)-3-(2,4,6-trichlorophenyl)thiourea hemihydrate (II)(Khawar Rauf *et al.*, 2009) and 1-(1,3-benzothiazol-2-yl)-3- (4-chlorobenzoyl)thiourea (III) (Yusof *et al.* 2009) except the substituent attached to the terminal nitrogen atom is 3-fluorophenyl instead of 2,4,6-trichlorohenyl or benzothiazole. There are two molecules in the asymmetric unit of (II). The dihedral angle between the two benzene rings in each molecule is 66.93 (8)° and 60.44 (9)°. On the other hand, the dihedral angles between the benzene ring and the benzothiaozole in (III) of 28.42 (8)° indicating the role of chlorine atom on the stablity of the compound.

The molecule (I) is discrete (Figure 1) and adopts a typical *trans*-*cis* configuration with respect to the position of 4-chlorobenzoyl and 3-fluorophenyl fragments respectively against the thiono group across their C—N bonds. The benzene rings and thiourea moiety are each planar with maximum deviation of 0.025 (1)Å for N2 atom from least square plane. The dihedral angles between the two benzene rings of 34.47 (7)° is smaller than that in (II) but close to (III). The central thiourea moiety (N1/C8/N2/S1) makes dihedral angles with the benzene (C1—C6) and (C9—C14)rings of 15.44 (6)° and 21.68 (6)° respectively. The bond lengths and angles are in normal ranges (Allen *et al.*, 1987). There are two intramolecular hydrogen bonds, N2—H2A..O1 and C14—H14A..S1, forming two pseudo-six member rings [O1..H2A/N2/C8/N1/C7] and [S1..H14A/C14/C9/N2/C8]. In the crystal structure, molecules are linked by intermolecular hydrogen bonds, N1—H1A..S1 and C2—H2B..O1 (symmetry code as in table 2) forming one dimensional chain along *b* axis (Figure 2).

### Experimental

4-chlorobenzoyl chloride (1.74 g, 0.01 mol) was mixed with an equimolar amount of ammonium thiocyanate (0.76 g,0.01 mol) and 3-fluoroaniline (1.11 g, 0.01 mol) in 50 ml dried acetone. The mixture was refluxed for 2 h. The light yellow solution was filtered and left to evaporate at room temperature. Colourless crystals were obtained after a few days (Yield 89.2%; m.p 458.2–459.7 K).

### Refinement

H atoms on the parent carbon atoms were positioned geometrically with C—H= 0.93 Å and N—H = 0.86Å and constrained to ride on their parent atoms with *U*_iso_(H)= 1.2*U*_eq_(parent atom).

### Crystal data

**Table:**

C_14_H_10_ClFN_2_OS	*F*(000) = 632
*M**_r_* = 308.75	*D*_x_ = 1.518 Mg m^−^^3^
Monoclinic, *P*2_1_/*c*	Melting point: 459 K
Hall symbol: -P 2ybc	Cu *K*α radiation, λ = 1.54178 Å
*a* = 8.5778 (1) Å	Cell parameters from 24507 reflections
*b* = 11.7584 (2) Å	θ = 5.0–72.7°
*c* = 13.4069 (2) Å	µ = 4.03 mm^−^^1^
β = 92.448 (2)°	*T* = 293 K
*V* = 1351.00 (3) Å^3^	Block, colourless
*Z* = 4	0.50 × 0.29 × 0.25 mm

### Data collection

**Table:**

Oxford Diffraction Xcalibur Eos Gemini diffractometer	2685 independent reflections
Radiation source: Enhance (Cu) X-ray Source	2628 reflections with *I* > 2σ(*I*)
graphite	*R*_int_ = 0.027
Detector resolution: 16.1952 pixels mm^-1^	θ_max_ = 72.7°, θ_min_ = 5.0°
ω scans	*h* = −9→10
Absorption correction: multi-scan (*CrysAlis PRO*; Oxford Diffraction, 2010)	*k* = −14→14
*T*_min_ = 0.238, *T*_max_ = 0.432	*l* = −16→16
33403 measured reflections	

### Refinement

**Table:**

Refinement on *F*^2^	Primary atom site location: structure-invariant direct methods
Least-squares matrix: full	Secondary atom site location: difference Fourier map
*R*[*F*^2^ > 2σ(*F*^2^)] = 0.032	Hydrogen site location: inferred from neighbouring sites
*wR*(*F*^2^) = 0.090	H-atom parameters constrained
*S* = 1.06	*w* = 1/[σ^2^(*F*_o_^2^) + (0.0565*P*)^2^ + 0.5662*P*] where *P* = (*F*_o_^2^ + 2*F*_c_^2^)/3
2685 reflections	(Δ/σ)_max_ < 0.001
181 parameters	Δρ_max_ = 0.31 e Å^−^^3^
0 restraints	Δρ_min_ = −0.27 e Å^−^^3^

### Special details

**Table:**

Experimental. Empirical absorption correction using spherical harmonics, implemented in SCALE3 ABSPACK scaling algorithm, CrysAlisPro (Oxford Diffraction, 2010)
Geometry. All e.s.d.'s (except the e.s.d. in the dihedral angle between two l.s. planes) are estimated using the full covariance matrix. The cell e.s.d.'s are taken into account individually in the estimation of e.s.d.'s in distances, angles and torsion angles; correlations between e.s.d.'s in cell parameters are only used when they are defined by crystal symmetry. An approximate (isotropic) treatment of cell e.s.d.'s is used for estimating e.s.d.'s involving l.s. planes.
Refinement. Refinement of *F*^2^ against ALL reflections. The weighted *R*-factor *wR* and goodness of fit *S* are based on *F*^2^, conventional *R*-factors *R* are based on *F*, with *F* set to zero for negative *F*^2^. The threshold expression of *F*^2^ > σ(*F*^2^) is used only for calculating *R*-factors(gt) *etc*. and is not relevant to the choice of reflections for refinement. *R*-factors based on *F*^2^ are statistically about twice as large as those based on *F*, and *R*- factors based on ALL data will be even larger.

### Fractional atomic coordinates and isotropic or equivalent isotropic displacement parameters (Å^2^)

**Table:**

	*x*	*y*	*z*	*U*_iso_*/*U*_eq_	
Cl1	0.68504 (4)	0.20726 (4)	0.98436 (3)	0.03536 (13)	
S1	1.21472 (5)	0.03202 (3)	0.43885 (3)	0.03022 (13)	
F1	1.42690 (13)	0.17249 (11)	0.12225 (8)	0.0484 (3)	
O1	1.06117 (13)	0.36258 (8)	0.58155 (8)	0.0294 (2)	
N1	1.06930 (14)	0.17096 (10)	0.55597 (9)	0.0236 (3)	
H1A	1.0313	0.1078	0.5764	0.028*	
N2	1.20911 (14)	0.26185 (10)	0.43716 (9)	0.0244 (3)	
H2A	1.1773	0.3206	0.4686	0.029*	
C1	0.94098 (17)	0.14201 (12)	0.75021 (11)	0.0247 (3)	
H1B	0.9950	0.0807	0.7247	0.030*	
C2	0.86286 (18)	0.12932 (13)	0.83788 (11)	0.0278 (3)	
H2B	0.8634	0.0600	0.8712	0.033*	
C3	0.78396 (17)	0.22191 (13)	0.87489 (10)	0.0255 (3)	
C4	0.78102 (18)	0.32590 (13)	0.82683 (11)	0.0284 (3)	
H4A	0.7276	0.3871	0.8530	0.034*	
C5	0.85897 (18)	0.33731 (12)	0.73923 (11)	0.0265 (3)	
H5A	0.8578	0.4069	0.7062	0.032*	
C6	0.93944 (16)	0.24564 (11)	0.69979 (10)	0.0216 (3)	
C7	1.02701 (16)	0.26641 (12)	0.60795 (10)	0.0226 (3)	
C8	1.16519 (16)	0.16253 (12)	0.47485 (10)	0.0220 (3)	
C9	1.29923 (16)	0.28669 (12)	0.35414 (11)	0.0245 (3)	
C10	1.35933 (19)	0.39669 (14)	0.34964 (13)	0.0330 (3)	
H10A	1.3439	0.4479	0.4012	0.040*	
C11	1.4424 (2)	0.42933 (16)	0.26764 (15)	0.0414 (4)	
H11A	1.4825	0.5027	0.2651	0.050*	
C12	1.46672 (19)	0.35550 (16)	0.19007 (14)	0.0398 (4)	
H12A	1.5221	0.3776	0.1352	0.048*	
C13	1.40591 (18)	0.24824 (16)	0.19719 (12)	0.0333 (4)	
C14	1.32205 (17)	0.21067 (13)	0.27676 (11)	0.0274 (3)	
H14A	1.2824	0.1371	0.2784	0.033*	

### Atomic displacement parameters (Å^2^)

**Table:**

	*U*^11^	*U*^22^	*U*^33^	*U*^12^	*U*^13^	*U*^23^
Cl1	0.0334 (2)	0.0521 (3)	0.02125 (19)	−0.00362 (16)	0.00978 (15)	−0.00270 (15)
S1	0.0401 (2)	0.01980 (19)	0.0323 (2)	−0.00030 (13)	0.01862 (16)	−0.00299 (13)
F1	0.0517 (6)	0.0658 (7)	0.0294 (5)	0.0014 (5)	0.0214 (4)	0.0013 (5)
O1	0.0390 (6)	0.0187 (5)	0.0315 (6)	−0.0022 (4)	0.0119 (5)	−0.0016 (4)
N1	0.0312 (6)	0.0176 (5)	0.0228 (6)	−0.0027 (5)	0.0098 (5)	−0.0013 (4)
N2	0.0294 (6)	0.0201 (6)	0.0243 (6)	−0.0004 (5)	0.0085 (5)	−0.0008 (4)
C1	0.0313 (7)	0.0213 (6)	0.0219 (7)	0.0031 (5)	0.0034 (5)	−0.0026 (5)
C2	0.0347 (8)	0.0268 (7)	0.0220 (7)	−0.0002 (6)	0.0024 (6)	0.0018 (5)
C3	0.0235 (7)	0.0366 (8)	0.0165 (6)	−0.0032 (6)	0.0030 (5)	−0.0035 (5)
C4	0.0304 (7)	0.0287 (7)	0.0263 (7)	0.0042 (6)	0.0051 (6)	−0.0075 (6)
C5	0.0328 (8)	0.0213 (7)	0.0256 (7)	0.0015 (6)	0.0039 (6)	−0.0029 (5)
C6	0.0237 (6)	0.0216 (6)	0.0194 (6)	−0.0008 (5)	0.0024 (5)	−0.0028 (5)
C7	0.0250 (7)	0.0205 (7)	0.0223 (7)	0.0001 (5)	0.0028 (5)	−0.0025 (5)
C8	0.0242 (6)	0.0217 (6)	0.0202 (6)	−0.0013 (5)	0.0037 (5)	−0.0009 (5)
C9	0.0206 (6)	0.0269 (7)	0.0262 (7)	0.0007 (5)	0.0031 (5)	0.0077 (5)
C10	0.0328 (8)	0.0265 (7)	0.0400 (9)	−0.0007 (6)	0.0041 (6)	0.0072 (6)
C11	0.0337 (8)	0.0351 (9)	0.0558 (11)	−0.0053 (7)	0.0077 (8)	0.0205 (8)
C12	0.0295 (8)	0.0516 (10)	0.0393 (9)	0.0025 (7)	0.0121 (7)	0.0224 (8)
C13	0.0264 (7)	0.0474 (9)	0.0267 (8)	0.0051 (7)	0.0072 (6)	0.0090 (7)
C14	0.0243 (7)	0.0327 (8)	0.0258 (7)	−0.0003 (6)	0.0062 (6)	0.0048 (6)

### Geometric parameters (Å, °)

**Table:**

Cl1—C3	1.7350 (14)	C4—C5	1.383 (2)
S1—C8	1.6691 (14)	C4—H4A	0.9300
F1—C13	1.360 (2)	C5—C6	1.396 (2)
O1—C7	1.2243 (18)	C5—H5A	0.9300
N1—C7	1.3778 (18)	C6—C7	1.4896 (19)
N1—C8	1.3949 (17)	C9—C14	1.390 (2)
N1—H1A	0.8600	C9—C10	1.395 (2)
N2—C8	1.3332 (18)	C10—C11	1.390 (2)
N2—C9	1.4125 (18)	C10—H10A	0.9300
N2—H2A	0.8600	C11—C12	1.377 (3)
C1—C2	1.386 (2)	C11—H11A	0.9300
C1—C6	1.393 (2)	C12—C13	1.370 (3)
C1—H1B	0.9300	C12—H12A	0.9300
C2—C3	1.385 (2)	C13—C14	1.384 (2)
C2—H2B	0.9300	C14—H14A	0.9300
C3—C4	1.382 (2)		
			
C7—N1—C8	128.98 (12)	O1—C7—N1	122.32 (13)
C7—N1—H1A	115.5	O1—C7—C6	121.72 (12)
C8—N1—H1A	115.5	N1—C7—C6	115.94 (12)
C8—N2—C9	130.76 (13)	N2—C8—N1	114.77 (12)
C8—N2—H2A	114.6	N2—C8—S1	128.04 (11)
C9—N2—H2A	114.6	N1—C8—S1	117.17 (10)
C2—C1—C6	120.71 (13)	C14—C9—C10	120.01 (14)
C2—C1—H1B	119.6	C14—C9—N2	123.68 (13)
C6—C1—H1B	119.6	C10—C9—N2	116.20 (14)
C3—C2—C1	118.74 (14)	C11—C10—C9	119.56 (16)
C3—C2—H2B	120.6	C11—C10—H10A	120.2
C1—C2—H2B	120.6	C9—C10—H10A	120.2
C2—C3—C4	121.87 (13)	C12—C11—C10	121.50 (16)
C2—C3—Cl1	119.29 (12)	C12—C11—H11A	119.2
C4—C3—Cl1	118.84 (11)	C10—C11—H11A	119.2
C5—C4—C3	118.83 (14)	C13—C12—C11	117.22 (15)
C5—C4—H4A	120.6	C13—C12—H12A	121.4
C3—C4—H4A	120.6	C11—C12—H12A	121.4
C4—C5—C6	120.77 (14)	F1—C13—C12	119.25 (15)
C4—C5—H5A	119.6	F1—C13—C14	116.73 (16)
C6—C5—H5A	119.6	C12—C13—C14	124.02 (17)
C1—C6—C5	119.09 (13)	C13—C14—C9	117.68 (15)
C1—C6—C7	123.35 (12)	C13—C14—H14A	121.2
C5—C6—C7	117.48 (13)	C9—C14—H14A	121.2
			
C6—C1—C2—C3	−0.4 (2)	C9—N2—C8—N1	−177.20 (13)
C1—C2—C3—C4	0.1 (2)	C9—N2—C8—S1	4.6 (2)
C1—C2—C3—Cl1	179.79 (11)	C7—N1—C8—N2	−7.1 (2)
C2—C3—C4—C5	0.1 (2)	C7—N1—C8—S1	171.32 (12)
Cl1—C3—C4—C5	−179.56 (11)	C8—N2—C9—C14	20.2 (2)
C3—C4—C5—C6	0.0 (2)	C8—N2—C9—C10	−163.45 (15)
C2—C1—C6—C5	0.5 (2)	C14—C9—C10—C11	−0.3 (2)
C2—C1—C6—C7	177.04 (13)	N2—C9—C10—C11	−176.72 (14)
C4—C5—C6—C1	−0.3 (2)	C9—C10—C11—C12	0.2 (3)
C4—C5—C6—C7	−177.01 (13)	C10—C11—C12—C13	−0.2 (3)
C8—N1—C7—O1	6.6 (2)	C11—C12—C13—F1	179.93 (15)
C8—N1—C7—C6	−172.23 (13)	C11—C12—C13—C14	0.3 (3)
C1—C6—C7—O1	−158.63 (14)	F1—C13—C14—C9	180.00 (13)
C5—C6—C7—O1	17.9 (2)	C12—C13—C14—C9	−0.3 (2)
C1—C6—C7—N1	20.2 (2)	C10—C9—C14—C13	0.3 (2)
C5—C6—C7—N1	−163.19 (13)	N2—C9—C14—C13	176.49 (13)

### Hydrogen-bond geometry (Å, °)

**Table:**

*D*—H···*A*	*D*—H	H···*A*	*D*···*A*	*D*—H···*A*
C14—H14A···S1	0.93	2.57	3.1865 (15)	124
N2—H2A···O1	0.86	1.91	2.6402 (16)	141
N1—H1A···S1^i^	0.86	2.68	3.4134 (13)	145
C2—H2B···O1^ii^	0.93	2.48	3.3717 (18)	160

Symmetry codes:  (i) −*x*+2, −*y*, −*z*+1; (ii) −*x*+2, *y*−1/2, −*z*+3/2.
